# Temporal windowing of recurrent sinusitis improves EHR-based immunodeficiency classification

**DOI:** 10.70962/jhi.20260047

**Published:** 2026-08-31

**Authors:** Aaron T. Chin, Rachel Mester, Veronica Tozzo, Alexis V. Stephens, Lisa A. Bastarache, Bogdan Pasaniuc, Manish J. Butte

**Affiliations:** 1 Division of Immunology, Allergy and Rheumatology, Department of Pediatrics, University of California, Los Angeles, Los Angeles, CA, USA; 2 UCLA Health Information Technology, UCLA Health, University of California, Los Angeles, Los Angeles, CA, USA; 3 Department of Computer Science, University of California, Los Angeles, Los Angeles, CA, USA; 4 Department of Human Genetics, University of California, Los Angeles, Los Angeles, CA, USA; 5 Department of Biomedical Informatics, Vanderbilt University Medical Center, Nashville, TN, USA; 6 Department of Genetics, University of Pennsylvania, Philadelphia, PA, USA; 7 Institute of Biomedical Informatics, University of Pennsylvania, Philadelphia, PA, USA; 8 Department of Microbiology, Immunology, and Molecular Genetics, University of California, Los Angeles, Los Angeles, CA, USA

## Abstract

Recurrent sinopulmonary infections are a hallmark of common variable immunodeficiency (CVID), yet electronic health records (EHR) struggle to distinguish multiple discrete infections from a single infection comprising multiple clinic visits. Using diagnosis codes and antibiotic prescriptions, we developed a temporal windowing methodology that groups related sinusitis encounters into episodes based on timing and antibiotic escalation patterns. We applied this approach to 79 CVID patients, 224 rituximab-treated patients with hypogammaglobulinemia, and 2,994 matched controls. Immunodeficient patients had more frequent sinusitis episodes and greater episode burden than controls. Windowed episode features, when combined with infection PheCodes, yielded the best-performing Ridge regression classifiers in both the CVID-only (AUC-ROC 0.787) and pooled CVID/RTX (AUC-ROC 0.724) models, outperforming PheCodes combined with simple encounter counts and PheCodes alone. In a held-out cohort of five patients with pre-diagnosis sinusitis data, the classifier correctly flagged 4 of 5 as high-risk. Quantifying recurrence and refractoriness of infections through temporal windowing improves EHR-based phenotyping of immunodeficiency.

## Teaser

Chin et al. develop a temporal windowing methodology that groups recurrent sinusitis encounters into discrete episodes in electronic health records. Applied to patients with CVID and rituximab-associated hypogammaglobulinemia, windowed episode features improve machine learning classification of undiagnosed immunodeficiency compared to simple encounter counting or diagnosis codes alone.

## Introduction

The diagnosis of inborn errors of immunity (IEI) is often delayed. Recurrence of sinopulmonary infections is one of the most common cues leading to diagnosis, but it can take years for these recurrent infections to be noticed (1, 2). Accumulation of recurrent infections in undiagnosed individuals is associated with poorer outcomes, survival, and quality of life (1, 2). Among IEIs, common variable immunodeficiency (CVID) is particularly challenging to diagnose due to its heterogeneous presentation, which can include common infections such as sinusitis (3, 4, 5).

Recurrent sinusitis is a well-documented warning sign of IEI, and many individuals with CVID present with recurrent sinopulmonary infections at diagnosis (6). Indeed, sinusitis has been shown to be a distinguishing feature in predictive models that detected CVID using structured electronic health record (EHR) data (7, 8, 9). However, merely identifying the presence of sinusitis in the EHR without integrating the aspect of recurrence or refractory disease may not be sufficient to distinguish CVID from individuals who are prone to sinusitis for reasons other than immunodeficiency (10). Improving the quantification of the number of episodes of sinusitis may improve phenotyping accuracy and enable better prediction of patients with underlying high-risk conditions.

The task of capturing recurrence and refractory infections in EHR data presents unique methodological challenges (11). ICD-10 codes inconsistently capture recurrence and refractoriness, and simple encounter counts cannot reliably distinguish new from refractory infections (11, 12, 13). In this work, we leveraged a multi-institutional CVID cohort and rituximab-treated (RTX) patients with secondary hypogammaglobulinemia to test a rules-based temporal windowing approach incorporating antibiotic prescription data. Temporal windowing captures clinically meaningful recurrence patterns that are not evident by simply counting encounters and improves EHR-based classification of undiagnosed immunodeficiency.

## Results

### Cohort creation and characteristics

**Figure 1.**
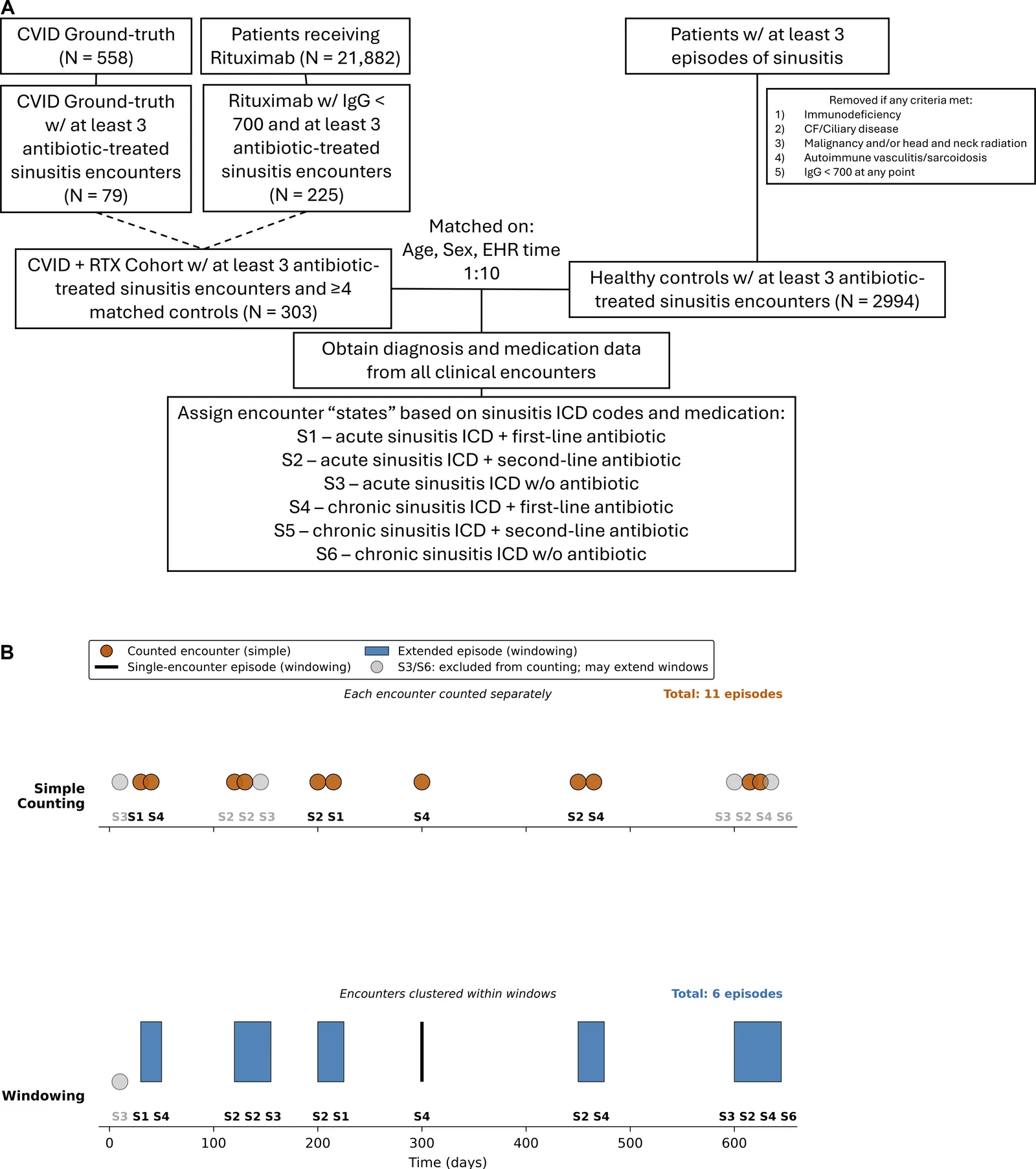
**Study cohort assembly and episode quantification methodology. (A)** CVID and RTX patients with hypogammaglobulinemia were filtered to ≥3 antibiotic-treated sinusitis encounters and matched 1:10 with controls on sex, age (±2 years), and EHR time (±180 days). Exclusion criteria included primary immunodeficiency (ICD-10 D80–D84), cystic fibrosis, structural airway abnormalities, autoimmune vasculitis, sarcoidosis, head and neck malignancy, and any measured IgG below 700 mg/dl. Encounter states (S1–S6) defined by sinusitis codes (J01, J32) and antibiotic type. **(B)** Example timeline: simple counting (11 episodes) versus windowing with 28-day search and 14-day extension windows (6 episodes). Orange circles, antibiotic-treated encounters (simple counting); gray circles, S3/S6 encounters excluded from counting; black lines, single-encounter episodes (windowing); blue bars, extended episodes (windowing).

We identified 558 CVID individuals confirmed by clinical immunologist review and 21,882 RTX patients (Fig. 1 A). After filtering for ≥3 antibiotic-treated sinusitis encounters, 79 individuals with CVID and 225 RTX-treated individuals met inclusion criteria. We built a matched control cohort based on sex, age at first encounter (±2 years), and total EHR length of observation (±180 days), allowing up to 10 control individuals per case. Controls required at least three antibiotic-treated sinusitis encounters and represent a sinusitis-enriched comparator, not the general population. One RTX individual was excluded after matching due to fewer than four available controls, yielding a final cohort of 79 CVID, 224 RTX-treated hypogammaglobulinemia cases, and 2,994 controls. Nine cases had partial matching (four to nine controls), while the remainder achieved full 1:10 matching. The final cohort had a median age of 63.0 years and was 39.9% male, with no significant differences in age or sex across groups, as these were matched.

### Episode definition using time-based windowing

**Figure 2.**
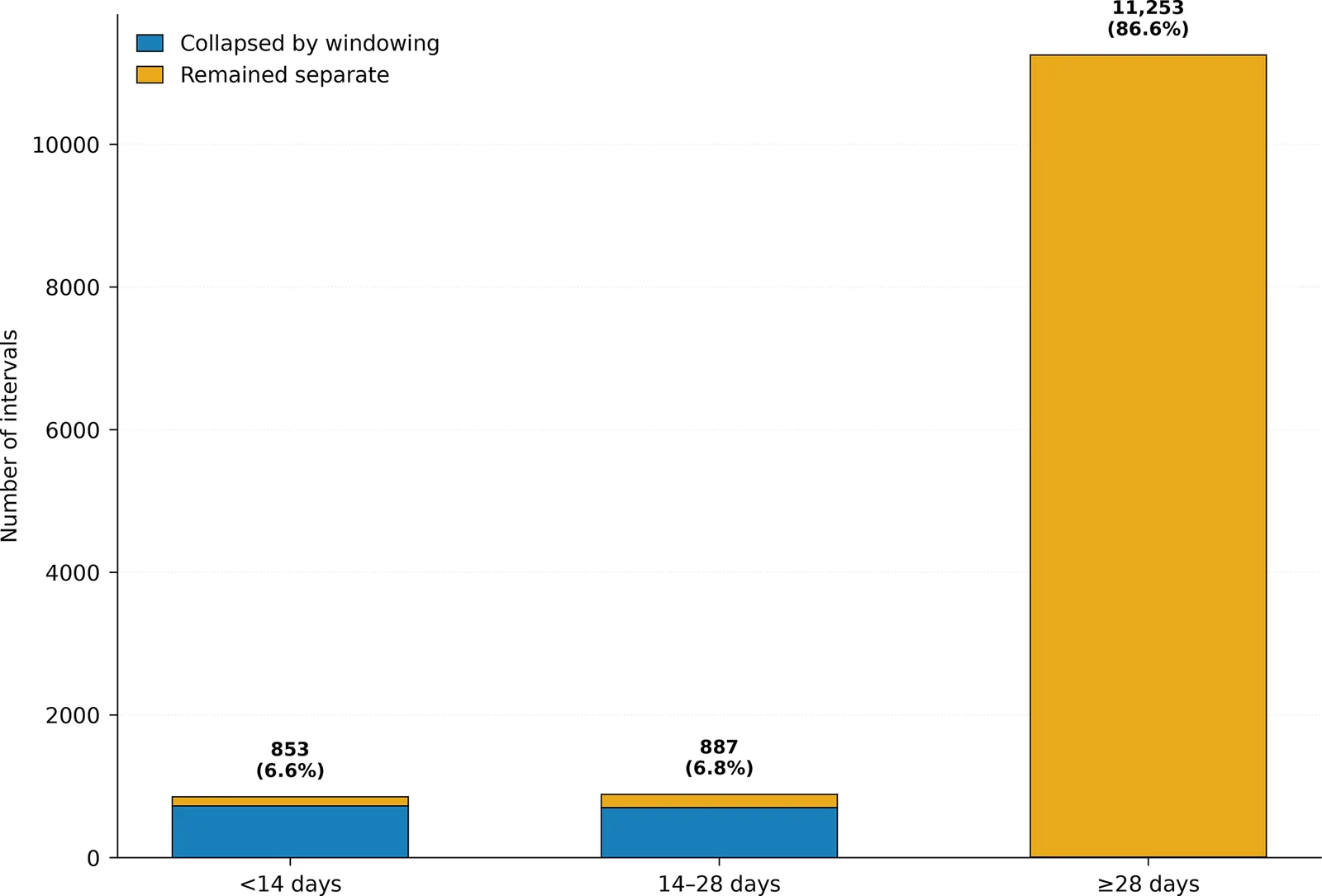
**Distribution of inter-encounter intervals and impact of windowing methodology.** Inter-encounter intervals grouped by length. Blue indicates intervals collapsed into an ongoing episode by windowing. Orange indicates intervals that remained separate. Windowing collapsed 1,435 of 12,993 intervals (11.0%), reducing the episode count from 16,008 to 14,624 (8.6%).

Simple counting sequentially numbered each antibiotic-treated encounter, while windowing methodology grouped temporally related encounters into episodes (Fig. 1 B). Of 12,993 total inter-encounter intervals, 853 (6.6%) were <14 days, and 887 (6.8%) fell within the 14–28-day window, where clinical ambiguity exists regarding whether a sinusitis encounter represents another visit from an ongoing sinusitis episode versus a new, independent episode. Most intervals (11,253, 86.6%) exceeded 28 days, representing independent episodes (Fig. 2). Windowing methodology collapsed 1,435 (11.0%) total inter-encounter intervals, reducing total episodes from 16,008 to 14,624 (8.6% reduction). The 313 intervals within 28 days that remained separate were encounters with non-specific ICD codes and no antibiotics, which could not extend existing episodes under windowing methodology rules.

### Immunodeficient patients exhibit greater sinusitis burden than controls

Individuals with CVID and RTX demonstrated greater sinusitis burden than controls (Table 1). CVID patients had more total episodes by simple counting than controls (5 [3, 8] vs. 4 [3, 5], P < 0.001) and by windowing (5 [3, 8] vs. 3 [3, 5], P < 0.001). Longest episode duration, mean encounters per episode, and episodes extended did not differ between CVID patients and controls (all NS). RTX patients had more episodes by simple counting than controls (4 [3, 7] vs. 4 [3, 5], P < 0.001) but not by windowing (NS). They showed greater mean encounters per episode, longer episode duration, and more extended episodes than controls (all P < 0.001). When CVID and RTX patients were combined, all episode metrics differed significantly from controls (all P < 0.001).

**Table 1.** Patient characteristics and sinusitis episode metrics by cohort

Variable	Total	CVID	RTX	Control	CVID vs. control	RTX vs. control	CVID+RTX vs. control
*N*	3,297	79	224	2,994	​	​	​
Age at end of follow-up (years)	63.0 [47.0, 73.0]	60.0 [47.0, 69.0]	64.0 [46.0, 74.0]	63.0 [46.0, 73.0]	​	​	​
Sex, male	1,317 (39.9%)	23 (29.1%)	99 (44.2%)	1,195 (39.9%)	​	​	​
Total episodes (simple counting)	4 [3, 5]	5 [3, 8]	4 [3, 7]	4 [3, 5]	<0.001	<0.001	<0.001
Total episodes (windowing)	3 [3, 5]	5 [3, 8]	4 [3, 6]	3 [3, 5]	<0.001	0.131	<0.001
Mean encounters per episode	1.0 [1.0, 1.2]	1.0 [1.0, 1.2]	1.2 [1.0, 1.4]	1.0 [1.0, 1.2]	0.513	<0.001	<0.001
Longest episode (days)	7 [7, 20]	7 [7, 26]	13 [7, 28]	7 [7, 18]	0.081	<0.001	<0.001
Episodes extended (%)	0.0 [0.0, 25.0]	0.0 [0.0, 19.4]	16.7 [0.0, 33.3]	0.0 [0.0, 25.0]	0.726	<0.001	<0.001

Continuous variables: median [IQR]; categorical variables: *n* (%). Pairwise comparisons used Mann–Whitney U (continuous) or Fisher’s exact test (categorical). The CVID+RTX vs. control column pools both immunodeficient cohorts against matched controls. Simple counting treats each antibiotic encounter as a separate episode; windowing groups related encounters using a 28-day initial window with a 14-day extension.

**Figure 3.**
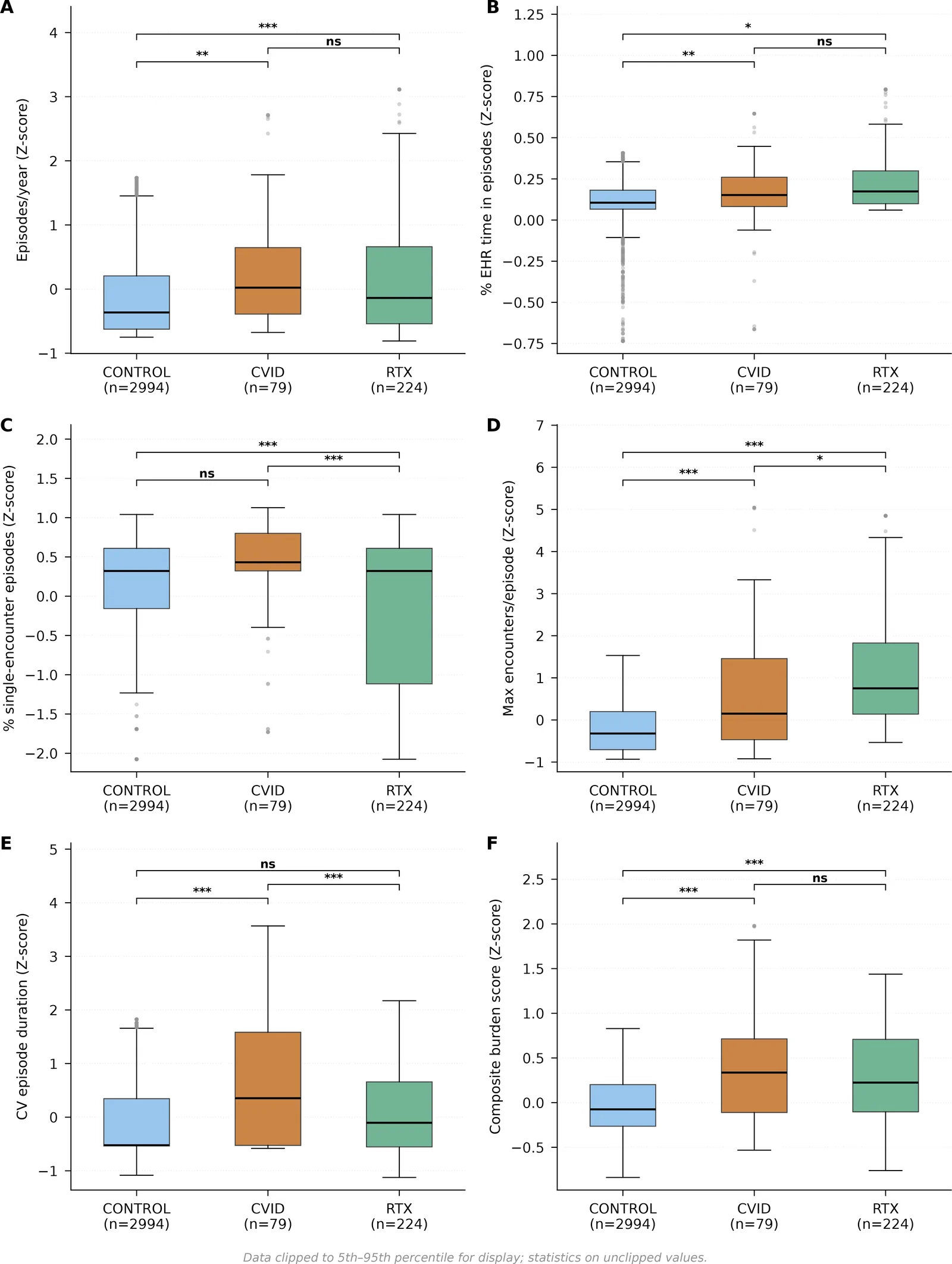
**Windowed episode features distinguish immunodeficient patients from controls. (A–F)** Z-normalized values for (A) episodes per year, (B) percent EHR time in episodes, (C) percent single-encounter episodes, (D) maximum encounters per episode, (E) coefficient of variation of episode duration, and (F) composite burden score (mean of panels A–E). Box plots show median (center line), interquartile range (box), and 1.5 × IQR whiskers. Significance: *P < 0.05; **P < 0.01; ***P < 0.001; ns, not significant (Welch’s *t* test). Display clipped to 5th to 95th percentile per group; statistics computed on full data.

Both CVID and RTX patients had higher episode frequency than controls (CVID: P = 0.003; RTX: P < 0.0001, Fig. 3 A). Both CVID and RTX groups also spent more time in episodes than controls (CVID: P = 0.004; RTX: P = 0.01, Fig. 3 B). CVID did not differ from controls in percent single-encounter episodes (NS), while RTX had a lower rate than controls and CVID patients (P < 0.001, Fig. 3 C). Both CVID and RTX had greater maximum encounters per episode than controls (CVID: P < 0.001; RTX: P < 0.001, Fig. 3 D), with RTX exceeding CVID (P = 0.03). CVID patients showed longer episode duration variability than both controls (P < 0.001, Fig. 3 E) and RTX (P < 0.001), while RTX did not differ from controls (NS). We then constructed a composite burden score by averaging the z-score–normalized features of five windowed episode features: episodes per year, percent EHR time in episodes, percent single-encounter episodes, maximum encounters per episode, and coefficient of variation of episode duration (see Materials and methods). The composite score (Fig. 3 F) was higher in CVID (0.46 ± 0.76) than controls (−0.03 ± 0.55, P < 0.001) with no difference between CVID and RTX (NS). RTX also exceeded controls (0.28 ± 0.72, P < 0.001).

### Windowed episode features combined with PheCodes outperform simple counting

**Figure 4.**
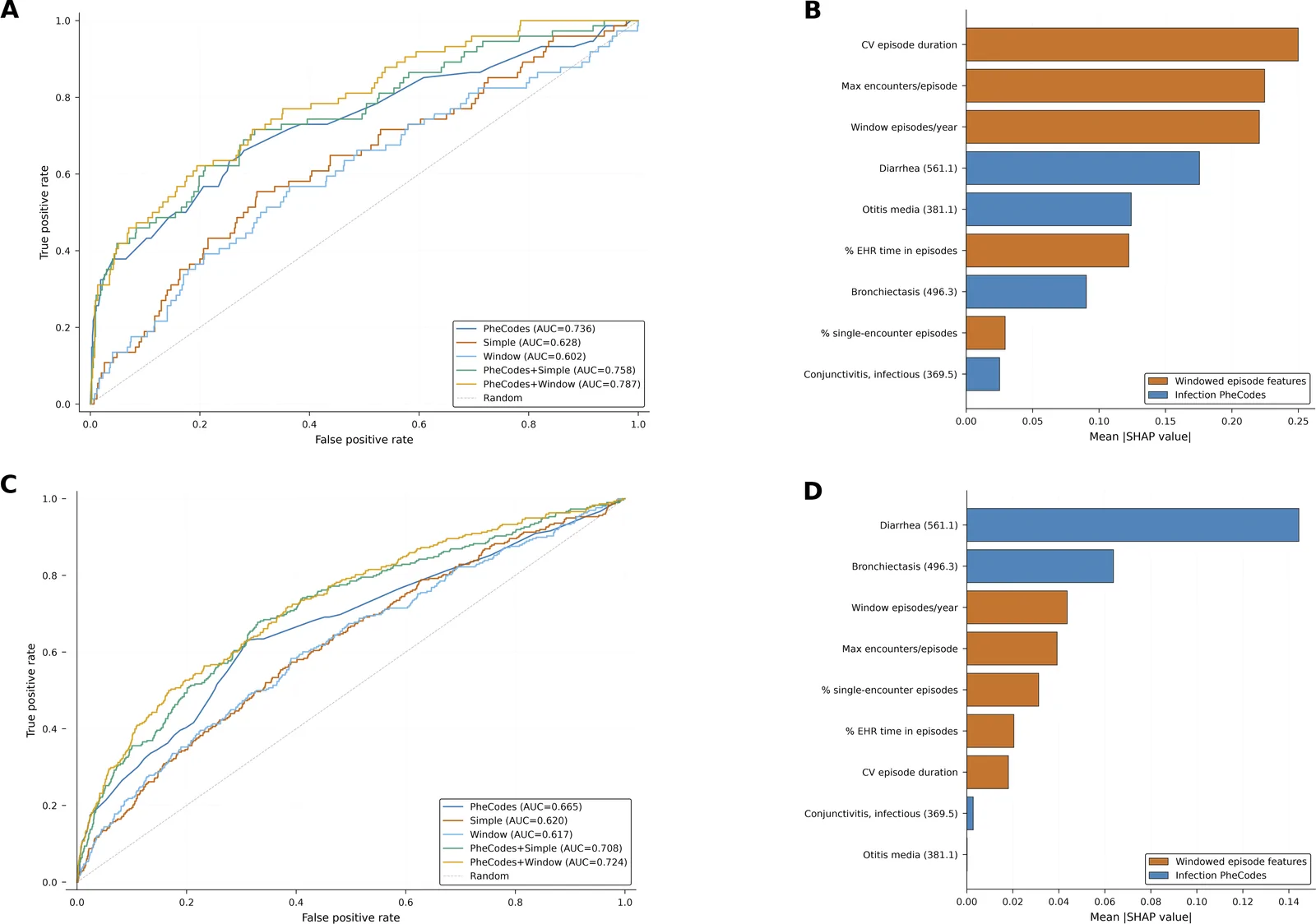
**Windowed episode features combined with PheCodes outperform simple counting and PheCodes alone in distinguishing CVID and CVID/RTX patients from controls. (A)** ROC curves from nested fivefold cross-validation showing out-of-fold performance for five Ridge regression classifiers distinguishing CVID patients from matched controls. **(B)** Mean absolute SHAP values for all features retained by the PheCodes + Window model after variance thresholding in the CVID-only analysis, ranked by importance. **(C)** ROC curves for the same five classifiers in the pooled CVID/RTX cohort. **(D)** Mean absolute SHAP values for features retained by the PheCodes + windowed episode features model in the pooled CVID/RTX cohort. Orange bars, windowed episode features; blue bars, infectious PheCodes. Pairwise AUC comparisons were performed using DeLong’s test with Bonferroni correction. PheCode numbers are shown in parentheses.

Five ridge regression classifiers were evaluated for distinguishing individuals with CVID from matched controls (Fig. 4 A). The PheCodes + windowed episode features model achieved the highest performance (AUC-ROC: 0.787, 95% CI: 0.730–0.839; AUC-PR: 0.388), outperforming PheCodes alone (AUC-ROC: 0.736, AUC-PR: 0.374), simple counting alone (AUC-ROC: 0.628, AUC-PR: 0.150), windowed episode features alone (AUC-ROC: 0.602, AUC-PR: 0.145), and PheCodes + simple counting (AUC-ROC: 0.758, AUC-PR: 0.373). The PheCodes + windowed episode features model significantly outperformed PheCodes + simple counting (DeLong’s test, Bonferroni-corrected P < 0.001). SHAP analysis identified coefficient of variation of episode duration (mean |SHAP|: 0.250), maximum encounters per episode (0.225), and windowed episodes per year (0.221) as the top three predictors of CVID status, all derived from windowing methodology, followed by diarrhea (0.176) and otitis media (0.124) among infection PheCodes (Fig. 4 B).

To increase statistical power and broaden the captured hypogammaglobulinemia phenotype, we added RTX patients with documented IgG below 700 mg/dl to the training set. We first verified that this population represented a clinically meaningful hypogammaglobulinemia phenotype rather than a malignancy- or autoimmune-driven artifact. RTX hypogammaglobulinemia patients met the more than three antibiotic-treated sinusitis criterion at higher rates than patients with the same conditions who did not receive rituximab, both in autoimmune indications (8.6 vs. 4.9%, P < 0.001) and lymphoma/leukemia indications (3.3 vs. 2.2%, P = 0.017) (Table S1). In the pooled CVID/RTX cohort, the PheCodes + windowed episode features model was the top-performing model (AUC-ROC 0.724, 95% CI: 0.697–0.758; AUC-PR: 0.277), as in the CVID-only trained models (Fig. 4 C). SHAP analysis of this pooled model identified diarrhea as the strongest predictor, followed by bronchiectasis, windowed episodes per year, maximum encounters per episode, and percent single-encounter episodes (Fig. 4 D).

A sensitivity analysis using varying search windows (14, 28, and 42 days) and extension windows (7, 14, and 21 days) was performed on both the CVID-only and pooled CVID/RTX models. AUC-ROC values ranged from 0.741 to 0.788 in the CVID-only model and 0.721 to 0.727 in the pooled CVID/RTX model across all nine parameter combinations (Table S2), indicating that model performance does not depend on precise tuning of episode definition parameters.

**Figure 5.**
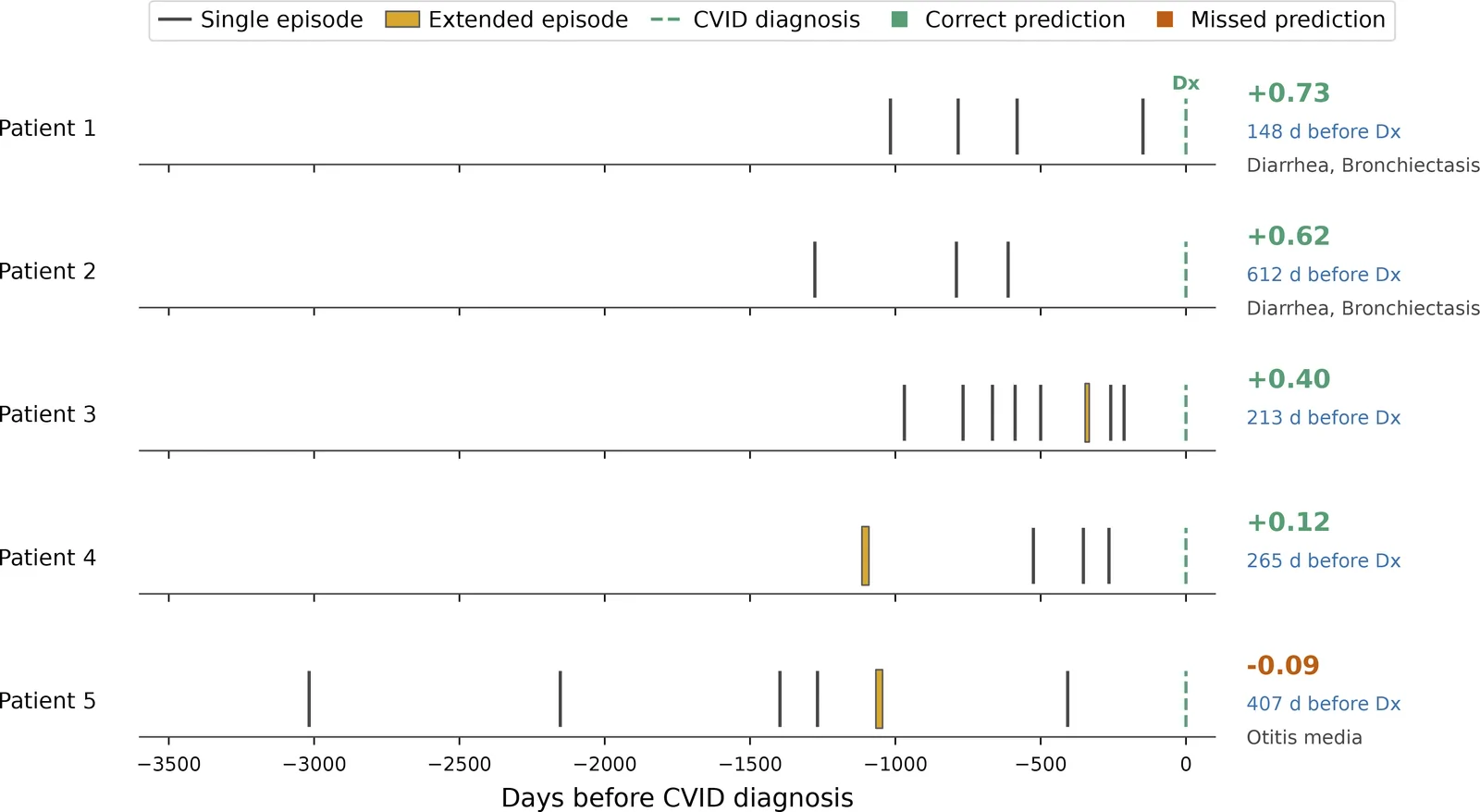
**Pre-diagnosis classification in a held-out CVID cohort.** Timeline of sinusitis episodes for five CVID patients using only pre-diagnosis data. Encounter states (S1–S6) labeled above each visit. Black lines indicate single-encounter episodes; amber bars represent extended episodes. Teal dashed line marks CVID diagnosis date. Risk scores from the pooled CVID/RTX PheCodes + windowed model are shown for each patient; teal score text indicates correct identification as high-risk; orange score text indicates a missed case. Patients 1–4 were correctly identified; the fifth was missed (80% sensitivity).

To evaluate classification performance on pre-diagnosis data, five CVID patients with more than three pre-diagnosis sinusitis episodes were excluded from model training and matched 1:10 with controls. Using only pre-diagnosis data, the PheCodes + windowed model trained on CVID/RTX patients correctly classified four out of five (80%) CVID patients as high-risk relative to healthy controls. Correctly identified patients had a mean gap of 310 days from last episode to diagnosis. Two patients did not have any predictive PheCode feature but were still predicted accurately. The CVID-only model, despite its higher discrimination on training data, identified three out of five patients in a pre-diagnosis cohort. Given the small sample size (*n* = 5), these results are exploratory evidence that windowed episode features combined with comorbid infection PheCodes retain signal on pre-diagnosis data, not validated evidence of early detection capability (Fig. 5).

## Discussion

Because the recurrence of infections has been a consistently highlighted “warning sign” in primary immunodeficiencies, we and others have used infectious phenotypes in developing EHR-based models to identify CVID among undiagnosed patients (7, 9). However, as much as these approaches have shown promise, none currently incorporates patterns of recurrence or refractory infections into their algorithmic scoring. Here, we demonstrate that a rule-based windowing methodology, which gathers individual encounters of infection into common episodes based on windows of time and escalation of antibiotics, is a viable approach to improve the prediction of individuals with humoral immunodeficiency. We trained two models, one using CVID versus controls and another using pooled CVID and RTX-induced hypogammaglobulinemia versus controls. In both, the use of windowed features significantly improved discrimination when combined with infection PheCodes from a previously trained model (9). This demonstrates that windowing methodologies can capture structured recurrence patterns not evident from simple encounter counts.

Epidemiologic studies have shown that infections concentrate within high-risk individuals who experience more frequent and longer episodes (11). Some ICD-10 codes include the terms “chronic” and “recurrent,” designed to denote temporal aspects beyond just the infection itself; however, their use is inconsistent (12, 13). Simple counts from claims data do not reliably distinguish new, recurrent, and refractory infections. Studies using ICD claim-free intervals and medication data have been used to approximate clinical episodes in other infectious diseases, but these approaches may misclassify follow-up visits as new infections (14, 15, 16, 17). In contrast, our approach captures refractory infectious episodes, which is a key feature of infectious episodes in immunodeficient patients. Our method uses clinician-defined thresholds (28-day search, 14-day extension) reflecting that patients returning within 28 days are likely to have a refractory case of sinusitis rather than a new or recurrent episode of sinusitis. This distinction resulted in features that improved model performance, showing that recurrent and refractory episodes are useful predictors for EHR-based prediction.

In the held-out pre-diagnosis cohort, classification accuracy reflected a combination of sinusitis episode burden and comorbid infection phenotype. Both models correctly classified most pre-diagnosis patients as high-risk using only data recorded before diagnosis. Although the CVID-only model achieved higher discrimination on training data, it classified three of five pre-diagnosis patients as high-risk, while the CVID/RTX-trained model classified four of five. The small sample size (*n* = 5) precludes definitive conclusions, and generalizability to a broader undiagnosed population requires validation in a larger, independent cohort.

Our study had several strengths. First, our CVID cohort comprises clinician-reviewed cases across multiple academic institutions. Second, we used structured EHR data (ICD codes, medications) that are portable and scalable. Because ICD codes have low sensitivity and high miscoding rates, we employed PheCodes and combined diagnoses with antibiotics to define episodes more accurately (12, 13). Prior work supports that medication data improve phenotyping, and this approach could extend beyond infections (e.g., steroids for autoimmune flares) (17, 18). Third, we expanded the case definition by incorporating RTX patients with hypogammaglobulinemia, increasing sample size for model training (19). Both groups share a phenotype of hypogammaglobulinemia and recurrent sinopulmonary infections, with sinusitis burden exceeding that of their underlying conditions alone rather than reflecting a malignancy- or autoimmune-driven artifact. Fourth, application of the trained model to a held-out pre-diagnosis cohort of CVID patients demonstrates that the classifier retains discriminative signal when restricted to data recorded before diagnosis. This analysis simulates a scenario in which the model is applied to a patient not yet diagnosed, though confirmation in a larger prospective cohort is needed before any claim of early detection capability.

There are several key limitations to our study. Windowed episode features require sufficient recurrent infections in temporal proximity to be informative. The inclusion criterion of three or more antibiotic-treated sinusitis encounters enriches for infection-predominant phenotypes, potentially underrepresenting patients with noninfectious CVID manifestations such as autoimmunity or granulomatous disease. This threshold is a study inclusion criterion, not a validated clinical trigger for diagnostic referral. Episode construction relies on structured codes and antibiotic prescriptions without chart-level validation. Requiring an antibiotic prescription to initiate an episode reduces, but does not eliminate the risk that follow-up visits or persistent diagnosis codes are misclassified as new infections. Prophylactic antibiotics suppress active infection signals and would not reflect true disease state in our episode framework. In this cohort, <0.2% of antibiotic-treated sinusitis encounters had a prescription duration consistent with prophylaxis. In populations with higher prophylactic antibiotic use, explicit dosing rules or clinical notes may be needed to distinguish prophylactic from therapeutic encounters. Many CVID patients in our ground-truth cohort were on immunoglobulin replacement, which may suppress sinusitis frequency and underestimate true episode burden. A model applied to undiagnosed, untreated patients would likely perform at least as well given the expected higher sinusitis burden in that population.

We restricted analysis to sinusitis, the most common infection in our cohort, as other infections occurred too infrequently for meaningful episode characterization. This study did not utilize unstructured data like clinical notes. Previous studies have shown recurrent infection can be mined from clinical text reliably (20), though it is important to note that clinical text is not always available to researchers and is not included in researcher accessible biobanks like UK Biobank and All of Us. We uniformly applied fixed rules-based thresholds (14-day extension, 28-day search window) across all patients. Sensitivity analysis across clinically plausible window parameters supported the use of these thresholds, though further work is needed to determine whether they generalize across all patient populations or infection contexts. Patients requiring longer clearance of infection, such as those with more severe immunodeficiency, may need longer windows than modeled here. Future approaches could use statistical methods to estimate patient-specific or context-specific episode boundaries by modeling infection duration distributions or incorporating factors that influence infection frequency and duration, such as age, disease severity, CVID phenotype, or seasonality (21).

Quantification of acute infectious episodes remains underexplored in EHR-based phenotyping. Simple rules-based windowing improves capture of recurrent and refractory episodes, enabling better discrimination of immunodeficient patients who exhibit greater infection burden. Future studies extending this methodology to other infections or acute autoimmune flares may further refine phenotype definitions and improve prediction models for complex immune-mediated conditions.

## Materials and methods

### Study cohort and data query

We conducted a retrospective cohort study using de-identified EHR data from the University of California health system, organized within the Observational Medical Outcomes Partnership common data model. The CVID cohort was curated by clinical immunologists across the UC system. A separate group of RTX patients with hypogammaglobulinemia was defined as patients with at least one IgG level below 700 mg/dl measured after their first rituximab dose.

Encounter data were extracted for all clinical encounter types. For each encounter, ICD-10 diagnostic codes, antibiotic prescriptions (ATC codes J01*), patient demographics, and age were extracted. Sinusitis encounters were identified using ICD-10 codes J01 and J32 (acute and chronic sinusitis), with ICD-9 equivalents (461.*, 465.*, and 473.*). Antibiotics were categorized into first-line therapy (amoxicillin-clavulanate) and second-line therapies (Table S3) based on typical acute sinusitis treatment guidelines. The case cohort was restricted to patients with at least three antibiotic-treated sinusitis encounters.

### Matched control curation

Controls were matched 10:1 to cases based on sex, age at first encounter (±2 years), and total EHR length (the difference between the last and first encounter date; ±180 days). Controls were restricted to patients with at least three antibiotic-treated sinusitis encounters. Individuals were excluded if they had diagnoses indicating primary immunodeficiency (D80–D84, ICD-9: 279.*), cystic fibrosis (E84, ICD-9: 277.0*), structural airway abnormalities (Q30–Q34, Q89.3, ICD-9: 748.*, 759.3), autoimmune vasculitis (M30.1, M31.3, M32, ICD-9: 446.4, 446.5, 710.0), sarcoidosis (D86, ICD-9: 135), head and neck malignancy (C00–C14, C30–C32, ICD-9: 140–149, 160–161), or any measured IgG below 700 mg/dl. Cases with fewer than four available matched controls were excluded from analysis.

### State definitions and episode quantification

Encounters were assigned to mutually exclusive states: S1 (acute sinusitis + first-line antibiotics), S2 (acute sinusitis + second-line antibiotics), S3 (acute sinusitis without antibiotics), S4 (chronic sinusitis + first-line antibiotics), S5 (chronic sinusitis + second-line antibiotics), and S6 (chronic sinusitis without antibiotics) (Fig. 1 A). Same-day encounters were collapsed using the hierarchy S1 > S4 > S2 > S5 > S3 > S6.

Two episode counting methods were applied (Fig. 1 B). Simple counting sequentially numbered all antibiotic-treated encounters (S1, S2, S4, and S5). Windowed episodes used a 28-day search window and 14-day extension window, based on the clinical expectation that patients returning within 28 days are likely to have refractory infection rather than new or recurrent sinusitis. Antibiotic-treated encounters (S1, S2, S4, and S5) initiated episodes by opening a 28-day “searching window.” Subsequent antibiotic-treated encounters (S1, S2, S4, and S5) or acute encounters without antibiotics (S3) extended the window search by an additional 14 days from that encounter. S6 encounters did not extend active episodes, as chronic sinusitis without prescribed antibiotics lacks sufficient specificity for active infection. Episodes closed when no extending encounters occurred within the active window. Treatment de-escalation from second-line (S2, S5) to first-line antibiotics (S1, S4) also could not extend episodes.

To assess potential prophylactic antibiotic use, we identified antibiotic-treated sinusitis encounters with a co-occurring prescription of 60 days or more. Fewer than 0.2% of encounters met this threshold, none in CVID patients. Given the low prevalence and the imprecision of duration as a surrogate for prophylactic intent, these encounters were retained in the analysis.

### PheCode mapping

ICD codes from encounters were mapped to PheCodes using PheCode Map 1.2 (22). Infection category PheCodes from a previously trained model to predict CVID were converted to binary patient-level features indicating presence (1) or absence (0) throughout the observation period (Table S4) (9).

### Feature engineering and disease burden assessment

Simple count of episodes tallied total antibiotic-treated encounters and calculated episode rate (episodes per year). Windowed episode features included: (1) episodes per year, (2) disease burden (percentage of observation time within active episodes), (3) episode simplicity (percentage of single-visit episodes), (4) worst episode intensity (maximum encounters within any episode), and (5) episode variability (coefficient of variation in episode durations). Maximum encounters per episode captures refractoriness within a single infection. Episode duration was defined as 7 days for single encounters, reflecting the typical antibiotic course duration. A composite burden score was calculated as the mean of five z-score–standardized windowed episode features. Group differences were evaluated using pairwise Welch’s *t* tests.

### Predictive modeling: CVID/RTX versus controls

We trained a logistic regression model with ridge regularization to distinguish CVID patients from matched controls. We used five feature sets: infection PheCodes alone (Table S4), simple counting alone, windowed episode features alone, infection PheCodes combined with simple counting, and infection PheCodes combined with windowed episode features.

Models used nested cross-validation with stratified fivefold outer CV for performance evaluation and fivefold inner CV for α selection. Class imbalance was addressed using balanced class weights. Features were z-score standardized within each training fold, and fold-constant features were removed using variance thresholding. Performance was assessed using out-of-fold AUC-ROC and average precision. Bootstrap 95% confidence intervals for AUC-ROC were computed from out-of-fold predictions. Model comparisons used DeLong’s test for pairwise ROC comparisons with Bonferroni correction (α = 0.005 for 10 comparisons).

To increase statistical power and broaden the captured hypogammaglobulinemia phenotype, we repeated the nested cross-validation pipeline with RTX patients with documented IgG below 700 mg/dl added to the case set, using identical feature sets and evaluation procedure. To assess whether RTX hypogammaglobulinemia represented a clinically meaningful phenotype rather than a malignancy- or autoimmune-driven artifact, we compared the rate of meeting the more than three antibiotic-treated sinusitis criterion between RTX and non-RTX patients with the same underlying indications, stratified into autoimmune and lymphoma/leukemia subgroups.

To evaluate robustness of the episode construction parameters, we performed a sensitivity analysis varying search windows of 14, 28, and 42 days and extension windows of 7, 14, and 21 days. For each of the nine parameter combinations, episode features were recomputed, and the PheCodes + windowed episode features model was re-evaluated using the same nested cross-validation procedure in both the CVID-only and pooled CVID/RTX models.

To evaluate pre-diagnosis classification performance, CVID patients with more than three pre-diagnosis sinusitis episodes were excluded from training and matched 1:10 with controls. The PheCodes + windowed features model was evaluated on its ability to distinguish these pre-diagnosis patients from controls.

### Online supplemental material

Supplemental material includes four tables. Table S1 reports sinusitis burden in RTX patients with hypogammaglobulinemia compared to non-RTX patients with the same underlying autoimmune or malignant indication, matched on age and EHR observation duration. Table S2 reports the window parameter sensitivity analysis for the PheCodes + windowed episode features model trained across nine combinations of search and extension window in both the CVID-only and pooled CVID/RTX cohorts. Table S3 lists the antibiotic classification scheme used for sinusitis episode state definitions. Table S4 lists all infection PheCodes tested in predictive models and indicates which were retained after variance thresholding.

## Ethics statement

This study was approved by the UCLA Institutional Review Board (IRB #20-2316). Informed consent was waived for this retrospective study using de-identified EHR data.

## Declaration of AI-assisted technologies

The authors used a large language model (Anthropic Claude, Opus 4.5 through 4.7) to assist with grammatical editing and sentence-level revision of manuscript text and to assist with plotting and figure assembly code. No manuscript sections were drafted de novo by AI, and no figure content was generated by AI; all visual elements represent real analysis output or author-created schematics. All output was reviewed and edited by the authors, who take full responsibility for the content of the publication.

## Supplementary Material

Table S1 shows sinusitis burden in RTX hypogammaglobulinemia versus non-RTX patients with the same underlying indication.

Table S2 shows window parameter sensitivity analysis.

Table S3 shows antibiotic classification for sinusitis episode definition.

Table S4 shows infection PheCodes tested and retained in predictive models.

## Data Availability

The datasets generated and analyzed during this study are not publicly available due to patient privacy protections and institutional data use agreements but are available from the corresponding author upon reasonable request and with appropriate data use agreements.
