# Supplementary material for: Temporal windowing of recurrent sinusitis improves EHR-based immunodeficiency classification
Source: J Hum Immun. 2026 Aug 31;2(6):e20260047. doi: 10.70962/jhi.20260047 (PMC13528580; doi:10.70962/jhi.20260047)
Supplement: Supplementary file 1 [file jhi_20260047_tables1.docx]

# Supplemental Materials

**Table S1. Sinusitis burden in rituximab-treated hypogammaglobulinemia versus non-rituximab-treated patients with the same underlying indication.**

|  | **Rituximab-treated, IgG <700 mg/dL** | **Not rituximab-treated** | **p-value** |
| --- | --- | --- | --- |
| **Autoimmune indications (n)** | 1,146 | 1,146 |  |
| % with any antibiotic-treated sinusitis | 22.6% | 17.0% | <0.001 |
| Meeting ≥3 sinusitis criterion | 8.6% | 4.9% | <0.001 |
| **Lymphoma or leukemia indications (n)** | 2,727 | 2,727 |  |
| % with any antibiotic-treated sinusitis | 14.3% | 9.8% | <0.001 |
| Meeting ≥3 sinusitis criterion | 3.3% | 2.2% | 0.017 |

*Rituximab-treated patients had hypogammaglobulinemia, defined as at least one IgG below 700 mg/dL after the first dose. Comparator patients had the same underlying indication and did not receive rituximab. The ≥3 sinusitis criterion is three or more antibiotic-treated sinusitis encounters. p-values by chi-square test*
