# Supplementary material for: Temporal windowing of recurrent sinusitis improves EHR-based immunodeficiency classification
Source: J Hum Immun. 2026 Aug 31;2(6):e20260047. doi: 10.70962/jhi.20260047 (PMC13528580; doi:10.70962/jhi.20260047)
Supplement: Supplementary file 2 [file jhi_20260047_tables2.docx]

**Table S2. Window parameter sensitivity analysis**.

| **Search window** | **Extension window** | **CVID-only AUC-ROC** | **Pooled CVID/RTX AUC-ROC** |
| --- | --- | --- | --- |
| 14 days | 7 days | 0.763 | 0.723 |
| 14 days | 14 days | 0.755 | 0.721 |
| 14 days | 21 days | 0.759 | 0.727 |
| 28 days | 7 days | 0.788 | 0.727 |
| **28 days** | **14 days** | **0.787** | **0.724** |
| 28 days | 21 days | 0.787 | 0.724 |
| 42 days | 7 days | 0.741 | 0.727 |
| 42 days | 14 days | 0.747 | 0.727 |
| 42 days | 21 days | 0.747 | 0.726 |

*Out-of-fold AUC-ROC for the PheCodes + windowed episode features model across nine combinations of search window and extension window, in the CVID-only and pooled CVID/RTX cohorts. The clinician-determined primary analysis parameters (search = 28 days, extension = 14 days) are bolded.*
