# Supplementary material for: Temporal windowing of recurrent sinusitis improves EHR-based immunodeficiency classification
Source: J Hum Immun. 2026 Aug 31;2(6):e20260047. doi: 10.70962/jhi.20260047 (PMC13528580; doi:10.70962/jhi.20260047)
Supplement: Supplementary file 3 [file jhi_20260047_tables3.docx]

**Table S3. Antibiotic Classification for Sinusitis Episode Definition**

| **Drug Name** | **Classification** |
| --- | --- |
| Amoxicillin-clavulanate | First-line |
| Cefdinir | Second-line |
| Cefpodoxime | Second-line |
| Cefuroxime | Second-line |
| Trimethoprim-sulfamethoxazole | Second-line |
| Azithromycin | Second-line |
| Clarithromycin | Second-line |
| Levofloxacin | Second-line |
| Moxifloxacin | Second-line |
| Ciprofloxacin | Second-line |
| Doxycycline | Second-line |
| Clindamycin | Second-line |
| Cefixime | Second-line |
| Metronidazole | Second-line |
| Cefadroxil | Second-line |
| Cefaclor | Second-line |
| Cephalexin | Second-line |
| Linezolid | Second-line |

*First-line therapy: amoxicillin-clavulanate. Second-line therapies: cephalosporins, macrolides, fluoroquinolones, and other agents used when first-line treatment fails or is contraindicated. Classification based on standard acute bacterial rhinosinusitis treatment guidelines.*
