# Supplementary material for: Temporal windowing of recurrent sinusitis improves EHR-based immunodeficiency classification
Source: J Hum Immun. 2026 Aug 31;2(6):e20260047. doi: 10.70962/jhi.20260047 (PMC13528580; doi:10.70962/jhi.20260047)
Supplement: Supplementary file 4 [file jhi_20260047_tables4.docx]

**Table S4. Infection PheCodes Tested and Retained in Predictive Models**

| **PheCode** | **Phenotype** | **Retained*** |
| --- | --- | --- |
| 475 | Chronic sinusitis | No |
| 480 | Pneumonia | No |
| 497 | Bronchitis | No |
| 41 | Bacterial infection NOS | No |
| 686 | Infections of skin and subcutaneous tissue | No |
| 561.1 | Diarrhea | Yes |
| 320 | Meningitis | No |
| 369.5 | Conjunctivitis, infectious | Yes |
| 381.1 | Otitis media | Yes |
| 465 | Acute upper respiratory infections | No |
| 496.3 | Bronchiectasis | Yes |
| 31 | Diseases due to mycobacteria | No |
| 472 | Chronic pharyngitis and nasopharyngitis | No |
| 136 | Other infectious and parasitic diseases | No |

**Retained after variance thresholding in final PheCodes+Window model. PheCodes with zero or near-zero variance across training data were removed prior to model fitting.*
